# Practice patterns of doctors of chiropractic with a pediatric diplomate: a cross-sectional survey

**DOI:** 10.1186/1472-6882-10-26

**Published:** 2010-06-14

**Authors:** Katherine A Pohlman, Maria A Hondras, Cynthia R Long, Andrea G Haan

**Affiliations:** 1 Palmer Center for Chiropractic Research, Palmer College of Chiropractic, Davenport, IA, USA; 2 Palmer College of Chiropractic, Clinic Assessment and Integrity, Davenport, IA, USA

## Abstract

**Background:**

Complementary and alternative medicine (CAM) is growing in popularity, especially within the pediatric population. Research on CAM practitioners and their specialties, such as pediatrics, is lacking. Within the chiropractic profession, pediatrics is one of the most recently established post-graduate specialty programs. This paper describes the demographic and practice characteristics of doctors of chiropractic with a pediatric diplomate.

**Methods:**

218 chiropractors with a pediatric diplomate were invited to complete our survey using either web-based or mailed paper survey methods. Practitioner demographics, practice characteristics, treatment procedures, referral patterns, and patient characteristics were queried with a survey created with the online survey tool, SurveyMonkey^©^^®^.

**Results:**

A total of 135 chiropractors responded (62.2% response rate); they were predominantly female (74%) and white (93%). Techniques most commonly used were Diversified, Activator ^®^, and Thompson with the addition of cranial and extremity manipulation to their chiropractic treatments. Adjunctive therapies commonly provided to patients included recommendations for activities of daily living, corrective or therapeutic exercise, ice pack\cryotherapy, and nutritional counseling. Thirty eight percent of respondents' patients were private pay and 23% had private insurance that was not managed care. Pediatrics represented 31% of the survey respondents' patients. Chiropractors also reported 63% of their work time devoted to direct patient care. Health conditions reportedly treated within the pediatric population included back or neck pain, asthma, birth trauma, colic, constipation, ear infection, head or chest cold, and upper respiratory infections. Referrals made to or from these chiropractors were uncommon.

**Conclusions:**

This mixed mode survey identified similarities and differences between doctors of chiropractic with a pediatric diplomate to other surveys of doctors of chiropractic, CAM professionals, and pediatric healthcare providers. The pediatric diplomate certificate was established in 1993 and provides didactic education over a 2 to 3 year span. The results of this study can be used for historical information as this specialty continues to grow.

## Background

Complementary and Alternative Medicine (CAM) describes a wide, heterogeneous range of approaches to prevent or treat diseases. The exact definition of CAM has been in considerable debate and has evolved over time[1]. The National Center for Complementary and Alternative Medicine (NCCAM) defines CAM as "a group of diverse medical and health care systems, practices, and products that are not generally considered to be part of conventional medicine[2]." NCCAM also divides CAM into mind-body medicine, biologically based therapies, manipulative and body-based systems, energy medicine, and whole systems approaches such as Ayurveda and Traditional Chinese Medicine[2]. In 2007, almost 4 out of 10 adults and 1 out of 9 children used a complementary and alternative medicine therapy[3]. Manipulation by either chiropractors or osteopaths was one of the most commonly used therapies (2.8%) by the pediatric population[3].

Most research on the use of CAM has focused on the patient perspective. Patient characteristics and reasons why CAM practitioners are sought have been identified[3-5]. Research is lacking about the actual practices of CAM practitioners and sub-specialties within those CAM practitioners, including doctors of chiropractic.

There are currently 17 accredited chiropractic colleges in the U.S. and each of these colleges has a curriculum which includes 4 to 5 academic years with a clinical internship. Licensure laws exist in all 50 U.S. states for doctors of chiropractic. All states also require a passing score in the National Board of Chiropractic Examiners (NBCE) and some states have additional examinations such as ethics and jurisprudence.

Within the chiropractic profession there are opportunities to obtain a clinical specialty, called a diplomate certification, in topics such as clinical neurology, sports chiropractic, nutrition, orthopedics, radiology, rehabilitation, and pediatrics. U.S. chiropractic colleges offer these programs through either part-time post-graduate continuing education courses or full-time residency programs. According to the 2005 Job Analysis of Chiropractic survey, 14% of practicing chiropractors hold diplomate status and an additional 22% stated that they have completed work toward diplomate status[6], but did not report what diplomate status respondents held. The pediatric post-graduate diplomate program was established in 1993. Graduates earn the pediatric diplomate through 180-360 hours of weekend courses over 2 to 3 years. The purpose of this survey was to describe the characteristics of doctors of chiropractic with a diplomate in pediatrics.

## Methods

In April 2009, we performed a cross-sectional survey of doctors of chiropractic who hold a diplomate in pediatrics. The Institutional Review Board at the Palmer College of Chiropractic approved this study.

### Study design

The survey was created using SurveyMonkey^©^^®^, an online survey tool (http://SurveyMonkey.com). SurveyMonkey allowed electronic self-administration and data collection as well as access to an exact paper replica of the survey. Participants accessed the survey from a website that introduced them to the study and was designed with quick links to information about the authors, frequently asked questions, references, and a link to request a paper version of the survey. Proper functioning of images and links and checks for clarity and content of our questionnaire were pre-tested using clinical research personnel at the Palmer Center for Chiropractic Research. Comments and suggestions arising from this pre-test were incorporated into the final version for study participants.

### Sample

The target population was the 218 doctors of chiropractic listed on the International Chiropractors Association specialty Council on Chiropractic Pediatrics (ICA-CCP)[7], the International Chiropractic Pediatric Association (ICPA)[8] and the Academy of Chiropractic Family Practice's (ACFP) websites as holding a diplomate in pediatrics[9].

### Data collection

An initial information letter explaining the purpose of the study and providing the uniform resource locator (url) to the website with the direct link to the survey was sent to each chiropractor. They were informed that the survey would take approximately 30 minutes to complete, that only a unique identification number would be accessible with their survey except for the project coordinator, that survey completion was voluntary, and that there were no incentives to complete the online survey. Consent was implied if they completed the survey. A follow-up letter was mailed 4 weeks later to all non-responders, followed by a paper version of the survey sent another 3 weeks later with a self-addressed stamped envelope.

The electronic survey was administered with a set sequence of twenty pages, the exact number of questions ranging from one to seven per page. The paper survey was twenty-six pages long, with the number of questions ranging from one to six per page. The electronic survey did not allow for blank responses, however respondents were given the option "Do not wish to answer" to refuse answering a question. Surveys that were started but not completed were used in the final analysis. We were not able to control for completeness with respondents completing the paper survey.

Information was collected about practitioner demographics, practice characteristics, treatment procedures, referral patterns, and patient characteristics. Because the survey was long we did not ask questions about diagnostic procedures. The questions were designed to compare our data with the job analysis of all doctors of chiropractic performed by the 2005 Job Analysis of Chiropractic Survey[6]. Types of conditions treated were also collected to compare with the conditions treated with complementary and alternative medicine described in the National Health Statistics Reports[3]. Data were analyzed using SPSS 15.0 (SPSS Inc, Chicago, IL).

## Results

### Response Rate

**Figure 1:**
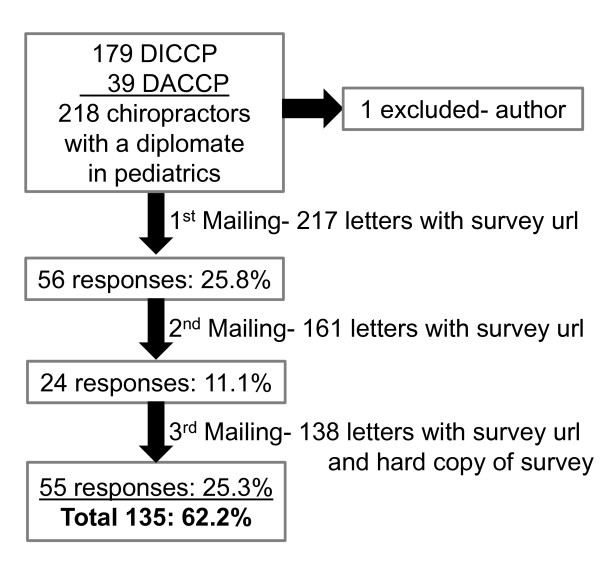
**Response rate for job analysis survey of chiropractors with a diplomate in pediatrics**.

As seen in Figure 1, 218 doctors of chiropractic were eligible to take part in this survey; one was excluded (author on the paper). One hundred and thirty-five responded for a 62% response rate. Of these, 102 respondents were from the ICA-CCP and 31 from the ICPA/ACFP. There were no practice pattern differences between the two organizations. From the respondents, 61% took the electronic survey and 39% the paper version.

### Demographics

Table 1 provides a summary of demographic characteristics. Respondents were predominantly female (74%) and white (93%). The majority were married (79%), worked 30-39 hours per week (41%), and treated 50-99 patients per week (32%). The average years in practice were 14.6 years (range 1-38). In addition to their chiropractic degree, 66% held a bachelor's degree and 6% held an advanced degree.

**Table 1:** Demographic data from doctors of chiropractic with a diplomate in pediatrics (n = 135)

	Total
Female- n (%)	103 (73.6)

Not Hispanic or Latino- n (%)	131 (93.6)

Race: White- n (%)	130 (92.9)

Marital Status- n (%)	

Married	111 (79.3)

Hours/Week spent working- n (%)	

≤ 10 hours	10 (7.1)

11-19 hours	15 (10.7)

20-29 hours	33 (23.6)

30-39 hours	58 (41.4)

40-49 hours	15 (10.7)

≥ 50 hours	4 (2.8)

Gross Annual Income- n (%)	

≤ $81,000	45 (32.1)

$81,000 - $200,000	53 (37.9)

≥ $200,000	27 (19.3)

Number of Patients Seen/Week- n (%)	

< 50	28 (20.0)

50-99	45 (32.1)

100-149	30 (21.4)

150-199	14 (10.0)

≥ 200	15 (10.6)

Size of community	

City	50 (35.7)

Suburb	44 (31.4)

Small Town	18 (12.9)

Rural	23 (16.4)

Years in Practice- mean (SD)	14.6 (7.61)

Highest non-chiropractic educ- n (%)	

Bachelor Degree	92 (65.7)

Higher Degree	8 (5.7)

Other Diplomate	

Yes	9 (6.4)

Working	13 (9.3)

Institution DC degree- n (%)	

Palmer College of Chiropractic- Davenport	65 (48.1)

Life University (Life College)	6 (4.4)

National University of Health Sciences	7 (5.2)

New York Chiropractic College	8 (5.9)

Northwestern Health Sciences University	8 (5.9)

Others	41 (30.4)

### Practice Characteristics

Sixty-four percent of the respondent's professional activities were spent in direct patient care. Record documentation and business management activities occupied 11% and 12% of their time, respectively. Fifty-two percent stated they participated in a managed care network, but only 22% of patient cases were paid through managed care. The majority of patient cases were handled with private pay/cash (30%) and private insurance (24%).

Most respondents were employed in a single practitioner (42%) or in a multi-chiropractor office (48%). Only 13% practiced in more than one location. Fifty-seven percent employed a chiropractic assistant and 71% stated they employed additional staff. Additional staff included receptionists, insurance clerks, office managers, massage therapists, radiology technicians, and acupuncturists. Chiropractic assistants were reported to perform the following tasks: x-ray development (33%), take x-rays (11%), obtain patient case history (8%), and perform adjunctive therapies (34%), although these procedures were reported for all patients, not pediatric patients alone. Forty percent of respondents indicated they had completed more than 30 hours of continuing education units during the previous year, the majority of which were attained by attending conferences/seminars (94%) and diplomate courses (60%).

### Treatment Procedures

#### Chiropractic Techniques

The most common chiropractic technique used by respondents was Diversified, which was used daily by 59% of these doctors. Diversified, a full spine technique, is taught at each chiropractic college[6] and is most often described as a high-velocity, low amplitude maneuver usually associated with a "popping" sound. Techniques used at least once every other week were Activator^®^, a mechanically-assisted chiropractic treatment device (63%) and Thompson, a drop-table assisted chiropractic treatment (59%), both of which may involve a thrust, but not typically resulting in joint cavitation. Respondents also reported the addition of cranial (77%) and extremity (77%) techniques to spinal manipulation. Additional techniques written in by respondents were: myofascial work (a technique that applies sustained pressure into the myofascial connective tissue restrictions), muscle testing (aka Applied Kinesiology, a concept from traditional Chinese medicine that is noninvasive and used to assess the body's imbalances), Webster technique (a technique aimed at reducing pelvic torsion in pregnant women), infant toggle (a specific upper cervical technique with a drop-head piece for assistance), sustained contact (a low-force, little to no amplitude technique in which a specific location is held for an extended amount of time), Bio-Geometric Integration (a technique that is incorporated with other chiropractic techniques to include a geometric model of the body and incorporating concepts of tensegrity, biodynamics, and force dynamics), and Neuro-Emotional Techniques (a mind-body technique aimed at evoking a somatic reflex).

The pattern of chiropractic techniques used differed some across patient age groups. Respondents most commonly used cranial techniques in their chiropractic treatment of patients five years and younger. Activator^® ^technique was used uniformly across all age groups, but Diversified and Thompson techniques were more commonly used in older patients.

#### Additional Therapies

Health promotions recommended by the doctor at least once every other week were breastfeeding recommendations and disease prevention/early screening advice. Other health promotion recommendations provided at least once per month included changing risky/unhealthy behaviors, ergonomic/postural advice, nutritional/dietary counseling, physical fitness/exercise, relaxation/stress reduction advice, and self-care strategies.

Adjunctive therapies provided at least every other week included recommendations for activities of daily living, corrective or therapeutic exercise, ice pack/cryotherapy, nutritional counseling, rehabilitation, and trigger point therapy. Therapies provided once a month included acupressure, electrical stimulation/therapy, foot orthotics/heel lifts, homeopathic remedies, hot pack/moist heat, massage therapy, mobilization therapy, taping/strapping, traction, and ultrasound. Several therapies (e.g. acupuncture with needles, biofeedback, paraffin bath) were never used. Additional reported therapies included laser light therapy, allergy testing, and brain balance therapy or neuromuscular reeducation.

### Referral Patterns

As shown in Table 2, referrals made to or from these chiropractors were uncommon. Massage therapists, midwives, family practitioners, and other chiropractors were the most common professionals referred to or by these doctors of chiropractic at least 1 to 10 times per year. Respondents also referred their patients to acupuncturists, ortho/neuro specialists and pediatricians. Other providers who referred to these doctors of chiropractic included a geriatrician, neurosurgeon, maternal health nurse, occupational therapist, radiologist, craniosacral therapist, optometrist, nurse practitioner, and naturopathic physician. Additional providers referred to included an occupational therapist, naturopathic physician, neurosurgeon, rolfer, hypnotherapist, optometrist, and exercise therapist.

**Table 2:** Mean referrals made to and from doctors of chiropractic with a diplomate in pediatrics (n = 132)

	Referrals made to chiropractors	Referrals made from chiropractors
Massage Therapist	1.2	2.1

Midwife	1.2	1.2

Family Practitioner	1.2	1.5

Other Chiropractor	1.2	1.2

Doula	0.9	0.9

Ob/Gyn	0.8	0.8

Acupuncturist	0.8	1.2

Dentist	0.7	0.9

Lactation Consultant	0.7	0.9

Pediatrician	0.7	1.1

Internist	0.6	0.8

Ortho/Neuro specialist	0.6	1.1

Physical Therapist	0.6	0.9

Nutritionist	0.5	0.8

Podiatrist	0.4	0.7

Therapeutic Yoga	0.4	0.8

Psychologist	0.3	0.6

Surgeon	0.3	0.6

Physiatrist	0.2	0.2

(scale: 0-Never; 1-Rarely; 2-Sometimes; 3-Often; 4-Routinely)

### Patient Characteristics

#### Patient Demographics

Survey respondents' characterizations of their patients are provided in Table 3. Twenty four percent of the respondents' patient populations were less than 5 years of age and 15% were between 5 and 18 years of age. Their patient populations were typically female (63%) and white (88%). Fifty-seven percent of their pediatric patients had a parent or primary caregiver who also received chiropractic care.

**Table 3:** Doctors of chiropractic with a diplomate in pediatrics characterization of their patients- mean percentage (SD) (n = 128)

	Total
Female	62.7 (12.64)

Age	

< 3 months	6.4 (6.81)

≥ 3 months to < 3 years	9.7 (8.77)

≥ 3 years to < 5 years	7.7 (5.35)

≥ 5 years to < 12 years	7.7 (5.73)

≥ 12 years to < 18 years	7.2 (4.63)

≥ 18 years to < 31 years	15.7 (10.03)

≥ 31 years to < 51 years	26.3 (14.57)

≥ 51 years to < 65 years	11.8 (7.65)

≥ 65 years	7.6 (6.64)

Hispanic	7.2 (12.29)

Race	

American Indian or Alaskan Native	0.9 (2.36)

Asian	3.9 (5.52)

Native Hawaiian or Other Pacific Islander	1.1 (3.25)

Black or African American	6.3 (8.35)

White	88.0 (11.88)

#### Patient Conditions

Respondents were asked to provide the frequency of care for conditions they treated during the past year in their patients less than 18 years of age. No specified condition was treated at a frequency of more than 1 or 2 times per week. The conditions with an average treatment frequency of 1 to 3 visits per month included back or neck pain, asthma, birth trauma, colic, constipation, ear infection, head or chest cold, and upper respiratory infections. Conditions treated 1-10 times per year included abdominal pain, ADHD/ADD, anxiety/stress, autism, depression, enuresis, infectious diseases, influenza or pneumonia, insomnia or trouble sleeping, nursing/suckling issues, parasites, respiratory allergy, scoliosis, sinusitis, and sore throat. Additional conditions reported by multiple providers of chiropractic were extremity complaints, headaches, acid reflux, torticollis, cranial distortions, wellness, growing pains, sports injuries, and traumatic injuries.

They also described the frequency of care for conditions they treated during the past year in patients 18 years and older. There were no conditions with a mean frequency of treatment more than 2 times per week. Conditions with a mean frequency of 1 or 2 times per week included back pain or problem, neck pain or problem, joint pain or stiffness, arthritis, regular headaches, severe headaches or migraines, sprain or strain, and stress. Conditions treated 1 to 3 times per month were anxiety, depression, fibromyalgia, head or chest cold, hypertension, insomnia or trouble sleeping, stomach or intestinal illness. Additional conditions reported included hormone issues, maternal concerns, extremity issues, allergies, constipation, and wellness care.

## Discussion

During the past two decades, there has been increasing interest in pediatrics in the chiropractic profession resulting in the development of two post-graduate pediatric diplomate programs. These programs were developed by doctors of chiropractic with clinical experience and success in treating pediatric patients. The first program, developed in 1993 by the International Chiropractors Association specialty Council on Chiropractic Pediatrics, offers a Diplomate in Clinical Chiropractic Pediatrics[7]. Currently it is administrated by the International Council on Chiropractic Pediatrics, which is not affiliated with any chiropractic professional association, but supported by both the International Chiropractors Association and the American Chiropractic Association. In 2002, the second program was established leading to a Diplomate in Pediatrics from the Academy Council of Chiropractic Pediatrics[9] organized by the International Chiropractic Pediatric Association[8].

Both pediatric diplomate programs consist of post-graduate weekend courses administered through a chiropractic college's post-graduate department. The weekend courses span 2 to 3 years and do not offer clinical rotations. Topics covered include all aspects of pediatrics from conception through birth, infancy, and adolescence. The goal is for practicing doctors of chiropractic to acquire greater skill and competency with the evaluation, diagnosis, and assessment procedures for the pediatric population, as well as to obtain manipulative therapy skills for this population and clinical conditions with which they present to a chiropractor. The two programs differ in amount of classroom hours (180 to 360 hours), number of examinations (2 orals and 2 written to 1 written), and additional requirements, such as scientific papers/presentations and participation in practice-based research networks. For both post-graduate diplomates, program requirements must be met before one is eligible to sit for their respective certification examination. Examinations are administered by the organization offering the program and are not currently governed by a regulatory body.

The National Board of Chiropractic Examiners (NBCE) performed a job analysis survey of doctors of chiropractic in 2003[6]. This was a random sample survey of US chiropractors who were in full-time practice. Responding chiropractors were white males (82%) who practice 30-39 hours per week (49%). Over half of their work time was devoted to direct patient care (52.9%). Patients presenting to these chiropractic offices were reportedly white females and between the ages of 31 to 64 (50.8%). Eight percent of the NBCE respondent patients were 5 years of age or younger and 10% were 6 to 17 years old.

Based on the names on the target sample list for our survey, we determined there were 79% females. Our survey was a direct comparison of many items of the NBCE survey and showed triple the number of female providers (74%) with a pediatric diplomate. Although this differs from the NBCE respondents (18% female[6]), it is similar to naturopathic medicine, another common CAM profession[10]. Chiropractors from our survey reported their patients are primarily female and white, similar to the NBCE survey. As expected, our respondents had a higher percentage of pediatric patients than the NBCE survey. Chiropractors in our survey spent about 63% of their time in patient care and saw approximately 100 patients per week, compared with the NBCE results of 53% of chiropractor's time spent in patient care[6].

In 2007, the National Health Statistics Reports (NHSR) used a multistage stratified design survey of the civilian, noninstitutionalized, household population in the United States. Some of the NHSR data were about CAM usage among adults and children[3], but they did not categorize the practice characteristics of CAM practitioners who treated children. The NHSR survey found that patients presented to manipulative/body based practices with a spectrum of complaints and a wide variety of conditions. The most common conditions reportedly treated in the pediatric population in our study (back or neck pain and head or chest colds) were the same as the NHSR 2007 CAM survey[3].

Birdee et al used the results from the 2007 NHSR and examined independent associations of CAM use with other factors, such as sociodemographic factors, prescription medication use, delays in health care caused by access difficulties, and common medical conditions/symptoms[11]. White individuals were more common than non-Hispanic black or Hispanic persons to seek manipulation and bodywork, which was similar to findings in our survey. Birdee et al also found that adolescents with musculoskeletal conditions, abdominal pain and nausea/vomiting used manipulation and bodywork. Chiropractors in our survey reported commonly treating pediatric patients with musculoskeletal conditions and occasionally abdominal pain, but not nausea/vomiting. We also observed that parental use of chiropractic was associated with the child use, consistent with Birdee et al findings with CAM in general.

In a survey of Danish chiropractic practices, Hestbaek et al found that children less than one year of age were the most common pediatric patients and chronic musculoskeletal pain was the most common complaint amongst the older children and adolescents[12]. These results were similar to the findings in our survey, as well as conditions reported in the NHSR survey.

Although the educational content of the diplomate programs emphasizes referral to pediatric providers, our survey did not show that this was common practice. This low referral pattern is consistent with both the NBCE survey and other chiropractic surveys[6,13]. In a recent national survey of pediatricians in the U.S., over 96% believed that their patients were using some sort of CAM therapy[14]. Sawni and Thomas provided a list of medical problems for which pediatricians referred or considered referring for CAM therapies including chronic problems (headaches, abdominal pain, asthma, pain management), behavioral problems, and neurological diseases (seizures, muscular dystrophy, and cerebral palsy)[14].

### Limitations of the study

In our attempt to collect a comprehensive description of this specialty in the chiropractic profession, the survey length may have inhibited participation. Additionally, by providing respondents with the option for mailing back a paper survey, we were unable to probe for missing data or incomplete responses.

### Future Directions

Each chiropractic school offers at least one pediatric course in the core curriculum and there are questions on the national board examination related to the pediatric population. However, the knowledge base and skill of the doctor of chiropractic to treat the pediatric population has not been investigated. Although this survey was not designed to assess the chiropractor skill set for treating pediatric patients, this may be an ideal next step along with assessing the content of the post-graduate examining boards' diplomate examinations.

The results of our survey indicate that providers of chiropractic with a pediatric diplomate treat a wide range of health problems. These results also provide a historical background of this specialty within the chiropractic profession that can be used to identify changes over time in response to increasing research into the safety, efficacy, and cost-effectiveness of chiropractic treatment for the pediatric population.

## Conclusions

This mixed mode survey is novel to the specialty of pediatrics within the chiropractic profession. The pediatric specialty is relatively new to the chiropractic profession and enthusiasm has prompted the formation of two diplomate certification programs. These programs provide didactic educational experience through weekend courses over the span of 2 to 3 years. The majority of chiropractors with a pediatric diplomate are females and likely use Diversified, Thompson, and Activator ^® ^techniques on their patients with the addition of cranial and extremity manipulation. They are more likely than other chiropractors to treat young patients who typically present with back or neck pain, head or chest colds, colic, constipation, ear infections and upper respiratory infections. The results of this study can be used as historical information as this specialty continues to grow, and may assist with the development of education of this post-graduate specialty within the chiropractic profession.

## Competing interests

The authors declare that they have no competing interests.

## Authors' contributions

KP conceptualized the overall project, designed and directed collection of the original data, analyzed the data, and drafted the manuscript. MH, CL, AH participated in the design of the overall project and manuscript, and quality control. All authors critically edited drafts of this manuscript and approved the final manuscript.

## Authors' information

KP has a DC degree and a diplomate in pediatrics. She is also a Clinical Research Fellow working on a Master of Science in Clinical Research at the Palmer Center for Chiropractic Research. Her graduate advisory committee is chaired by MH and also includes CL and AH. MH also has a DC and a MPH in epidemiology and is an associate professor at the Palmer College of Chiropractic. CL has a PhD in biostatistics and is a professor at the Palmer Center for Chiropractic Research. AH has a DC and a MS degree, and is the Director of Clinical Assessment and Integrity at Palmer College of Chiropractic.

## Pre-publication history

The pre-publication history for this paper can be accessed here:

http://www.biomedcentral.com/1472-6882/10/26/prepub
